# Comprehensive Assessment of Medial Knee Joint Instability by Valgus Stress MRI

**DOI:** 10.3390/diagnostics11081433

**Published:** 2021-08-09

**Authors:** Malin Ciba, Eva-Maria Winkelmeyer, Justus Schock, Philipp Schad, Niklas Kotowski, Teresa Nolte, Lena Marie Wollschläger, Matthias Knobe, Andreas Prescher, Christiane Kuhl, Daniel Truhn, Sven Nebelung

**Affiliations:** 1 Department of Diagnostic and Interventional Radiology, Aachen University Hospital, 52074 Aachen, Germany; malin.ciba@rwth-aachen.de (M.C.); eva.winkelmeyer@rwth-aachen.de (E.-M.W.); pschad@ukaachen.de (P.S.); niklas.kotowski@rwth-aachen.de (N.K.); tnolte@ukaachen.de (T.N.); ckuhl@ukaachen.de (C.K.); dtruhn@ukaachen.de (D.T.); 2 Department of Diagnostic and Interventional Radiology, Medical Faculty, University Düsseldorf, 40225 Düsseldorf, Germany; justus.schock@med.uni-duesseldorf.de (J.S.); lena.wollschlaeger@med.uni-duesseldorf.de (L.M.W.); 3 Department of Orthopaedic and Trauma Surgery, Cantonal Hospital Lucerne, 6000 Lucerne, Switzerland; matthias.knobe@luks.ch; 4 Institute of Anatomy, RWTH Aachen University, 52074 Aachen, Germany; aprescher@ukaachen.de

**Keywords:** knee joint, magnetic resonance imaging, functionality, biomechanics, valgus stress, medial collateral ligament, anterior cruciate ligament

## Abstract

Standard clinical MRI techniques provide morphologic insights into knee joint pathologies, yet do not allow evaluation of ligament functionality or joint instability. We aimed to study valgus stress MRI, combined with sophisticated image post-processing, in a graded model of medial knee joint injury. To this end, eleven human cadaveric knee joint specimens were subjected to sequential injuries to the superficial medial collateral ligament (sMCL) and the anterior cruciate ligament (ACL). Specimens were imaged in 30° of flexion in the unloaded and loaded configurations (15 kp) and in the intact, partially sMCL-deficient, completely sMCL-deficient, and sMCL- and ACL-deficient conditions using morphologic sequences and a dedicated pressure-controlled loading device. Based on manual segmentations, sophisticated 3D joint models were generated to compute subchondral cortical distances for each condition and configuration. Statistical analysis included appropriate parametric tests. The medial compartment opened gradually as a function of loading and injury, especially anteriorly. Corresponding manual reference measurements by two readers confirmed these findings. Once validated in clinical trials, valgus stress MRI may comprehensively quantify medial compartment opening as a functional imaging surrogate of medial knee joint instability and qualify as an adjunct diagnostic tool in the differential diagnosis, therapeutic decision-making, and monitoring of treatment outcomes.

## 1. Introduction

Alongside the anterior cruciate ligament (ACL), the superficial medial collateral ligament (sMCL) is one of the most often injured structures of the human knee joint [1,2]. Epidemiologic studies have reported the prevalence of isolated ACL and sMCL injuries and their combination as approximately 20%, 8%, and 2%, respectively, of all knee injuries undergoing specialist treatment [3]. Secondary to sports activities of young individuals that involve contact or cutting maneuvers, excessive valgus stress is the most common mechanism of injury [4,5]. Timely and correct diagnosis is essential to correctly identify the grade of injury and to plan treatment accordingly, both in the case of isolated and combined sMCL injuries [6,7].

While providing the basis of any diagnostic work-up, clinical testing of medial joint stability by application of valgus loads in extension and 30° of flexion is inherently subjective, affected by pain tolerance, muscle relaxation, and the examiner’s clinical experience, and particularly equivocal in combined injuries [8,9]. Consequently, its diagnostic value is only moderate in non-specialist settings [10]. Alternative diagnostic options involve valgus stress radiography, stress sonography, and magnetic resonance imaging (MRI).

Stress radiography and fluoroscopy under valgus loading quantify the medial compartment’s extension as a surrogate marker of sMCL injury [11,12]. Clinical adoption is limited because of multiple drawbacks and pitfalls such as the exposure to ionizing radiation, the acquisition of mere 2D projection radiographs, the limitations in reliability and reproducibility, the variability in positioning and loading protocols, and the unstandardized image analysis and quantification procedures [11,13,14].

Stress sonography has been under decade-long clinical investigation [15]. Despite its operator-dependence and reliance on high-resolution equipment, stress sonography is a promising and useful diagnostic tool if used properly and adopted by more joint surgeons and radiologists [16,17].

MRI is the superordinate diagnostic imaging modality for suspected ligament injuries of the knee because of its soft tissue contrast, spatial resolution, non-invasiveness, and lack of radiation [18,19,20]. After acute trauma, clinical (i.e., statically acquired) MRI studies indicate sMCL tears with sufficiently high sensitivity, yet low specificity. In particular, differentiating partial from complete sMCL tears and multiple-ligament injuries is diagnostically challenging [20,21,22]. Additional problematic constellations involve MCL bursitis, soft tissue cellulitis, medial meniscal cysts, and reactive fluid accumulations due to osteoarthritis, medial meniscus tears, meniscocapsular separations, and other soft tissue pathologies [18]. In chronic settings, the sMCL is thickened and may demonstrate altered signal characteristics because of calcification or ossification [19]. The sMCL’s morphologic appearance may thus not always correlate with its function.

While sMCL tears are usually treated conservatively and have favorable outcomes, some indications for surgical treatment are important to detect for appropriate patient management [9]. These include entrapment of the torn sMCL and distal or high-grade sMCL tears (i.e., grade III) if combined with ACL tears, even though treatment algorithms and timelines remain controversial [7,23,24].

In consideration of these diagnostic limitations of clinical MRI, the present study’s objective was to further improve the diagnosis of medial knee joint injuries. To this end, clinical MRI sequences were complemented by sophisticated image post-processing and pressure-controlled valgus loading in an in-situ model of graded sMCL and ACL injuries. We hypothesized that (i) increasing severity and extent of medial knee joint injuries are reflected by abnormal femorotibial responses to valgus loading as surrogates of joint instability and that (ii) these alterations can be accurately quantified using automatic and manual measurements of medial compartment opening. Prospectively, once normative values of medial compartment opening have been defined as a function of loading and injury, valgus stress MRI may be used to improve grading of medial joint instability through visualizing the injury and its functional consequences and to guide treatment and monitor return of stability during treatment or rehabilitation.

## 2. Materials and Methods

### 2.1. Study Design and Sample Size Estimation

This study was designed as a prospective experimental in-situ imaging study on human cadaveric knee joint specimens and performed between October 2020 and February 2021 in accordance with the relevant local guidelines and regulations. Local Institutional Review Board approval (Ethical Committee, RWTH Aachen University, EK180/16 issued 13 July 2016) and written informed consent by the body donors were obtained beforehand. Power analyses on the initial three knee joints (power, 0.8; probability of type-I-error, 0.05; effect size, 1.6; two-tailed test; www.statstodo.com, accessed on 12 November 2020) indicated a minimum sample size of eight.

### 2.2. MRI-Compatible Loading Device

**Figure 1:**
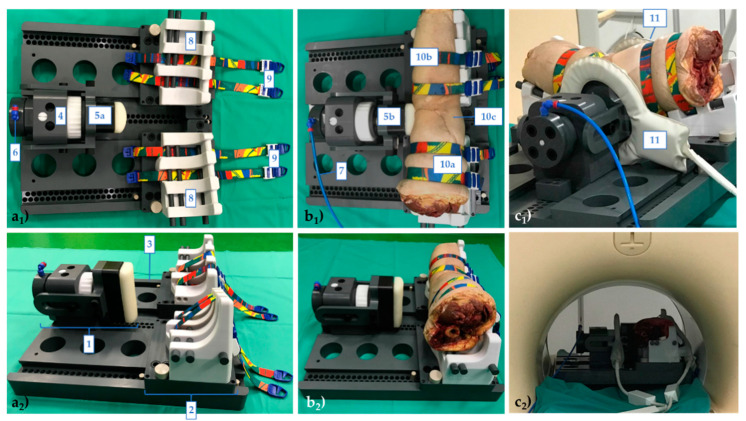
Experimental setup used for in-situ imaging of knee joints under valgus loading. Top (**a_1_**,**b_1_**) and side views (**a_2_**,**b_2_**) of the loading device without (**a**) and with (**b**) a representative (right-sided) knee joint. The loading device consists of a loading (1) and holding unit (2) that are both attached to the rigid base plate (3). The pneumatic unit (4) actuates the padded load applicator (5a—not pressurized, 5b—pressurized) which is aligned along the joint line and connected to the in-house pressure supply via a pressure port (6) and respective pressure lines (7). The holding unit standardizes joint position at 30° of flexion by two quarter-pipe wedges (8). Fixation is provided by two tourniquets (9) at the level of the thigh (10a) and lower leg (10b), while the popliteal fossa (10c) is not fixed. Fully operational setup with two flexible dual-coils positioned in lateral orientation to the knee (11) outside (**c_1_**) and inside (**c_2_**) a clinical 3.0T-MRI scanner.

For this study, a pressure-controlled MRI-compatible loading device developed and validated earlier [25,26] was used and further modified. Consisting of a loading, holding, and control unit, the device had been designed along the leverage principle with a padded load applicator providing the fulcrum around which the thigh and lower leg pivoted when loaded (Figure 1). Emulating the clinical valgus stress test [27], the pneumatic mechanism displaced the load applicator against the lateral joint line, thereby exerting distraction forces on the medial femorotibial compartment. Joint position was standardized by two equally sized quarter-pipe wedges of 15° inclination each, i.e., in 30° of joint flexion. Loading-induced compensatory motion was prevented by four tourniquets (CBC tourniquet, Kimetec GmbH, Ditzingen, Germany) made of MRI-compatible polyester and elastane. Pressure was provided by the in-house pressure supply, transferred to the device via standard pressure lines, and regulated by the control unit located outside of the MRI scanner room by means of a digital-to-analogue converter, an electronic pressure valve, and customized software routines implemented in LabVIEW software (National Instruments, Austin, TX, USA).

### 2.3. Human Cadaveric Knee Joint Specimens

Eleven human right knee joint specimens (fresh and unfixed, 3 women and 8 men) were provided through the local Department of Anatomy (RWTH Aachen University, Aachen, Germany) by body donors who had deceased due to unrelated medical conditions at a mean age of 74.5 ± 13.5 years (range, 48–95 years).

### 2.4. Pre-Imaging Preparations and Image Acquisition

After setting up the MRI-compatible loading device (Figure 1a), the knee joints were tightly fixed at the thigh and lower leg with two tourniquets each (Figure 1b). The knee joints were oriented with the patella facing downwards, equivalent to a patient’s prone position. For imaging, a clinical 3.0T scanner (Achieva, Philips, Best, The Netherlands) and two flexible multi-purpose phased-array transmit-and-receive dual-coils (Sense Flex-M, Philips) were used. The coils were positioned around the load applicator (lateral) and along the wedges (medial) (Figure 1c). Imaging was performed in the unloaded and loaded configurations. For the unloaded configuration, the load applicator was brought in loose contact with the joint, while for the loaded configuration, the pressure level was set to 3.23 bar (equaling 147.2 N or 15 kp) [25]. Special attention was paid to exactly align the load applicator with the lateral joint line. After loading (and prior to imaging), an equilibration period of 5 min was observed. Following scout views, the imaging protocol consisting of proton density-weighted fat-saturated, T1-weighted, and T2-weighted 2D turbospin echo sequences (Table 1) was completed for each joint and configuration. Scanning was performed at room temperature.

Any pre-existent ligament injury was excluded. During the initial scan of the specimens, i.e., unloaded and intact, structural integrity of the collateral and cruciate ligaments, the quadriceps and patellar tendons, the posteromedial and posterolateral corners, and joint capsule was evaluated by SN (clinical radiologist, 8 years of experience in musculoskeletal imaging). Consequently, three specimens had been discarded because of signs of partial or complete injury of any of these structures.

### 2.5. Graded Knee Joint Ligament Injuries

**Figure 2:**
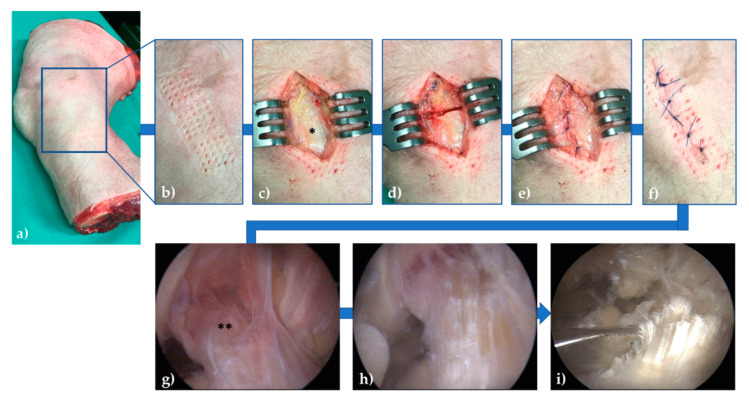
Graded knee joint ligament injuries via surgical (**b**–**f**) and arthroscopic (**g**–**i**) approaches. After identification of the medial joint line (**a**), the superficial medial collateral ligament (sMCL) was repetitiously stitched (*n* = 75 stitches) above, below, and at the level of the joint line (**b**). Then, via a longitudinal incision, the skin, subcutaneous tissue, and fascia were cut and retracted to expose the sMCL (*, **c**), which was completely transected at the femoral portion (**d**). Layer-wise closure of the superficial fascia (**e**) and the subcutaneous tissue and skin (**f**) was performed. Arthroscopically, the anterior cruciate ligament (ACL) was identified (**) (**g**), synovectomized (**h**), and completely transected at mid-substance level (**i**).

In between imaging, the knee joints were subject to sequential injuries via surgical and arthroscopic approaches outside the MRI. The sMCL was sequentially needled and transected which was followed by the additional transection of the ACL. Practically, the medial joint line was identified by palpation (Figure 2a). Using an MRI-compatible 5 × 5 needle block (Invivo BBC, Grid Biopsy System, Noras MRI products, Höchberg, Germany) and a standard 14 G needle of 1.75 mm diameter (Vasofix Braunüle, B.Braun Melsungen, Melsungen, Germany), the sMCL was needled multiple times along its proximodistal course. In total, each sMCL was subject to 75 needle stitches that were evenly distributed above (*n* = 25), below (*n* = 25) and at joint line level (*n* = 25) (Figure 2b).

Following a 4 cm-longitudinal skin incision, the subcutaneous fat and the superficial fascia were cut and retracted to expose the sMCL (Figure 2c), which was completely transected horizontally at the femoral portion using standard surgical scalpels (#10, Feather Safety Razor, Osaka, Japan) (Figure 2d). Subsequent wound closure was performed by layer-wise suturing of the fascia (Figure 2e), subcutaneous tissue and skin (Figure 2f) using polypropylene sutures (Prolene 4.0, Ethicon, Somerville, NJ, USA). In this experimental model, needle stitches were intended to emulate numerous microscopic fibre tears, while complete transection of the sMCL corresponded to grade-III sMCL injuries, i.e., complete ligament disruption [28].

Standard knee joint arthroscopy to transect the ACL was performed by SN (common trunk-trained orthopedic surgeon). The joint was accessed through the anteromedial and anterolateral portals. Then, the ACL was identified (Figure 2g), cautiously synovectomized for full visualization using a 4.5-mm full radius shaver (Dyonics, Smith & Nephew, Andover, MA, USA) (Figure 2h), and completely cut at its mid-substance using arthroscopic straight-tip scissors (Arthrex, Naples, FL, USA) (Figure 2i). Last, any excess fluid was removed, and the skin incisions were sutured, too.

The knee joints underwent imaging in terms of complete MRI series in the unloaded and loaded configurations prior-to, in-between, and after the sequential injuries. Consequently, imaging was performed in the (i) intact, (ii) partially sMCL-deficient, (iii) completely sMCL-deficient, and (iv) completely sMCL- and ACL-deficient conditions. Attention was paid to standardize the imaging and loading framework for each joint and configuration so that the orientation and dimension of the acquired MR sequences were exactly matched.

**Figure 3:**
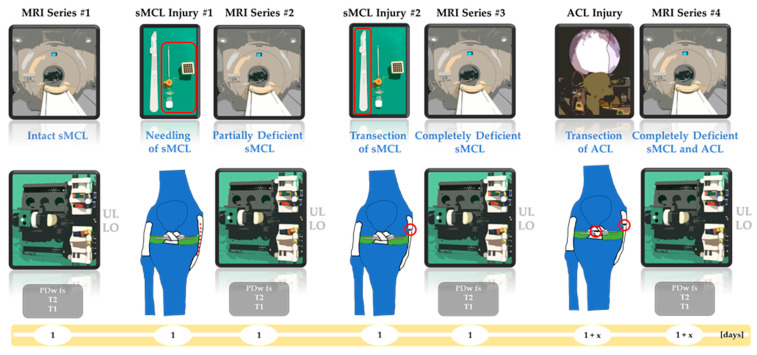
Timeline of MRI series and graded injuries. Sequentially, the superficial medial collateral ligament (sMCL) was partially injured, i.e., needled (sMCL injury #1), and completely injured, i.e., transected (sMCL injury #2). The anterior cruciate ligament (ACL) was transected (ACL injury). Consequently, the knee joints were imaged in the sMCL-intact condition (MRI series #1), partially sMCL-deficient condition (MRI series #2), completely sMCL-deficient condition (MRI series #3), and the combined sMCL- and ACL-deficient condition (MRI series #4). MRI series refer to the imaging protocol (grey boxes) that was completed for each joint in the unloaded (UL) and loaded (LO) configurations. Unit of timeline is days (yellow).

Figure 3 details the exact timeline of the MRI series and graded injuries.

Magnet time per joint, condition, and configuration was approximately 35 min. Total magnet time per knee joint was approximately 4.25 h. For logistical reasons, all joints were frozen once after MRI series #3 and thawed thoroughly before the arthroscopic ACL transection and MRI series #4. Sequential measurements were completed within 36 h. In-between the MRI series, the joints were kept refrigerated at 4 °C.

### 2.6. Image Post-Processing and Analysis

Femorotibial changes were evaluated for each joint, configuration (i.e., unloaded and loaded), and condition (i.e., intact, partially sMCL-deficient, completely sMCL-deficient, and completely sMCL- and ACL-deficient) based on manual 2D and computed 3D measurements.

#### 2.6.1. Manual 2D Reference Measurements

Following training on three knee joints under the guidance of SN, two readers (MC and EMW, medical students in their pre-graduate year) performed the manual reference measurements using the in-house picture archiving and communication system (iSite, Philips Healthcare, Amsterdam, The Netherlands) and its standard image analysis features.

**Figure 4:**
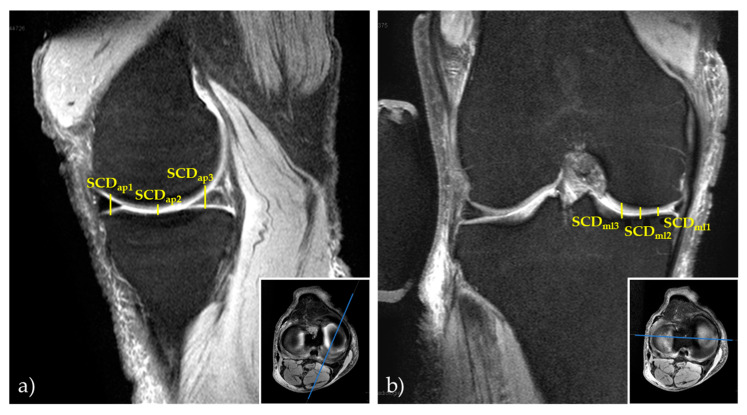
Example manual 2D reference measurements of subchondral cortical distances in the medial compartment. Visualized are the mid-sagittal (**a**) and mid-coronal (**b**) images of the Proton Density-weighted fat-saturated sequences and inset boxes to indicate their orientation. Subchondral cortical distances are indicated by yellow lines as the vertical distances between the femoral and tibial subchondral cortices at three locations along the anteroposterior (SCD_ap1_ to SCD_ap3_ [**a**]) and mediolateral dimensions (SCD_ml1_ to SCD_ml3_ [**b**]).

First, the mid-sagittal and mid-coronal images (with respect to the medial compartment) were identified on the Proton Density-weighted fat-saturated sequences. Second, the vertical distances between the tibial and femoral subchondral cortices (subchondral cortical distances [SCD]) were determined as surrogate markers of the medial joint space at three anteroposterior and three mediolateral locations on the mid-sagittal and mid-coronal images. More specifically, the locations were (from anterior to posterior) the centers of the anterior horn, body region, and posterior horn of the medial meniscus and (from medial to lateral) the peripheral, central, and internal intersections following the division of the medial tibial condyle into quarters (Figure 4). These locations are referred to as SCD_ap1_ to SCD_ap3_ (from anterior to posterior) and as SCD_ml1_ to SCD_ml3_ (from medial to lateral).

As loading configuration and joint condition were easily discerned, readers were not blinded, yet both readers were unaware of the other reader’s measurements. Inter-reader agreement was determined using the intraclass-correlation coefficient (online calculator v1.5, Mangold International, Arnstorf, Germany).

#### 2.6.2. Computed 3D Measurements

**Figure 5:**
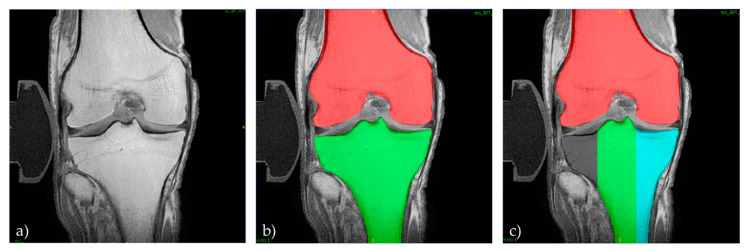
Manual segmentation procedure. For each joint, configuration, and condition, the coronal images of the T1-weighted sequence (**a**) were manually segmented and labelled as femur (red) and tibia (green) (**b**). The tibia was further compartmentalized into the medial (turquoise) and lateral (grey) tibial condyles and the intercondylar eminence was spared (**c**).

For each joint, configuration, and condition, the femoral and tibial bone outlines were manually segmented by MC based on the coronal T1-weighted sequences and the semiautomatic segmentation function of ITK-SNAP (v3.8, Cognitica, Philadelphia, PA, USA; [29]) as described before [26]. Additionally, the medial and lateral tibial condyles were labelled separately while sparing the intercondylar eminence to differentiate the medial and lateral compartments (Figure 5). For standardized definition of the medial and lateral tibial condyles, the height of the intercondylar eminence was determined as the vertical distance from its apex (i.e., most proximal extension) to its base (i.e., tangent along the joint line of the medial and lateral tibial condyles). The height of the intercondylar eminence was quartered, and the coordinates along the base quarter were then used to define the mediolateral borders of the intercondylar eminence.

Individual segmentation outlines were reviewed for accuracy and consistency by SN. Automatic pre-processing and harmonization procedures were implemented in Python (v3.6.5, Python Software Foundation, Wilmington, DE, USA) to realize consistent segmentation outlines. Connected-component analysis was performed to automatically discard all voxels that were not in contact with the manual segmentation outlines and to automatically label all voxels that were located within the segmentation outlines but had not been included.

Based on the segmentation outlines, 3D knee joint models were implemented for each joint, configuration, and condition, and used to automatically compute the SCDs in the medial compartment. Determination of SCDs was based on Cartesian coordinate systems along the three principal axes (Supplementary Figure S1). Initially, the input segmentations were converted to segmentation hulls at the relevant bone ends, i.e., the surfaces of distal femur and proximal tibia. Then, regularly spaced grids (set at intervals of 3.3 mm in anteroposterior and 3.5 mm in mediolateral direction) were defined on the hull for the medial and lateral tibial plateau. Originating from each grid point, grid lines were computed parallel to the z-axis and orthogonal to the x- and y-axes. Consequently, the vertical distances between the intersections of the grid lines and the femoral and tibial hulls were computed and converted to mm based on the voxel size of 0.36 × 0.36 × 3.0 mm^3^. Depending on the knee joint’s size, a mean of 85 ± 13 (mean ± standard deviation) individual SCD measurements were performed per medial compartment. To avoid erroneous SCD measurements at a distance to the surface of the distal femur, caused by the convex configuration of the medial femoral epicondyle, the medialmost (i.e., peripheral) grid points were excluded *à priori*. To this end, the joint’s maximum diameter along the transepicondylar axis was determined and all grid points within the medialmost 7% (of the maximum diameter) were discarded.

For intra- and inter-individual comparisons, the grid traces of all 3D joint models were registered to the grid trace of a selected average sized joint model in the unloaded configuration and intact condition.

The medial compartment’s 3D geometry was quantified for each joint, configuration, and condition by determining (i) the mean SCD over the medial compartment and (ii) the SCD at select anteroposterior and mediolateral locations analogous to the 2D reference measurements. To this end, corresponding 3D grid points and 2D locations were manually identified as individual measurements to undergo statistical analysis as detailed below.

### 2.7. Statistical Analysis

Statistical analyses were performed by MC and SN using GraphpadPrism (v9.1, San Diego, CA, USA). For the manual 2D reference measurements, excellent inter-reader agreement between both readers was determined with intraclass-correlation coefficients close to 1, i.e., 0.99 (SCD_ml1_), 0.98 (SCD_ml2_), 0.98 (SCD_ml3_), 0.97 (SCD_ap1_), 0.99 (SCD_ap2_), and 0.97 (SCD_ap3_) (single scorings, not adjusted). Consequently, both readers’ measurements were pooled for each location. Normal distribution of SCD values was confirmed using the D’Agostino Pearson test. Comparative analysis of computed 3D versus manual 2D measurements of SCD values at single locations was performed using the paired Student’s *t*-test. SCD values were longitudinally evaluated at single locations as a function of loading and condition using repeated measures two-way ANOVA with the joint as the subject and the factors condition and configuration as the repeating measures. Pairwise post-hoc comparisons were performed using Tukey’s test. Throughout, multiplicity-adjusted *p*-values are indicated to account for multiple comparisons against the family-wise alpha error threshold of *p* ≤ 0.01. A stricter-than-usual threshold was chosen to contain the number of statistically significant, yet clinically (most likely) insignificant findings.

## 3. Results

All 11 knee joints underwent sequential MR imaging in all configurations and conditions.

**Figure 6:**
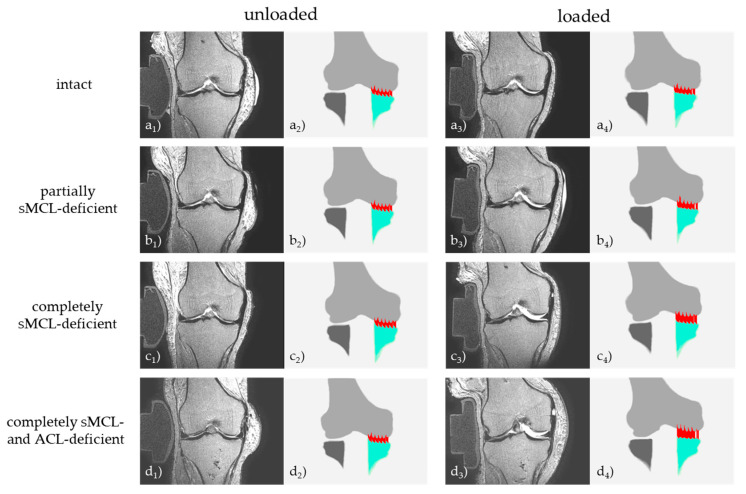
Loading-induced changes in the medial compartment as a function of graded knee joint ligament injury in a representative knee joint. Displayed are T2-weighted mid-coronal images (**a_1_**–**d_1_**,**a_3_**–**d_3_**) and corresponding (coronal) visualizations of the 3D knee joint models (**a_2_**–**d_2_**,**a_4_**–**d_4_**) in the unloaded (**a_1_**–**d_1_**,**a_2_**–**d_2_**) and loaded (**a_3_**–**d_3_**,**a_4_**–**d_4_**) configurations and the indicated joint conditions. Color codes are as follows: femur (light grey), tibia (turquoise [medial compartment] and dark grey [lateral compartment]). Vertical red lines indicate individual measurements of subchondral cortical distances. sMCL—superficial medial collateral ligament. ACL—anterior cruciate ligament.

Figure 6 visualizes example morphologic images and corresponding SCD measurements as a function of loading and condition.

**Figure 7:**
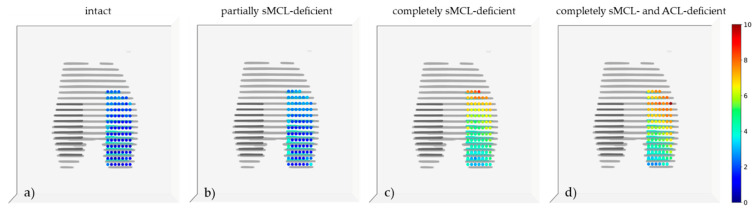
Spatially resolved quantification of medial compartment opening under loading as a function of graded knee joint ligament injury in all knee joints. Displayed are the loading-induced differences in the medial compartment’s subchondral cortical distances (SCD) for the unloaded versus loaded configurations and as a function of joint condition, averaged over all knee joints. (**a**) Intact, (**b**) partial sMCL deficiency, (**c**) complete sMCL deficiency, (**d**) combined sMCL and ACL deficiency. For example, “intact” visualizes the changes of the SCD values of the loaded versus the unloaded configuration. Axial views. Each color-coded dot represents a single SCD measurement along the pre-specified grid. The unit of the continuous colour scale on the right is [mm]. The sliced appearance is due to interslice gaps following axial image reconstruction. Abbreviations and color codes of bone outlines as in Figure 6.

Figure 7 plots the SCD values (as imaging surrogates of medial compartment opening) in a spatially resolved manner.

**Figure 8:**
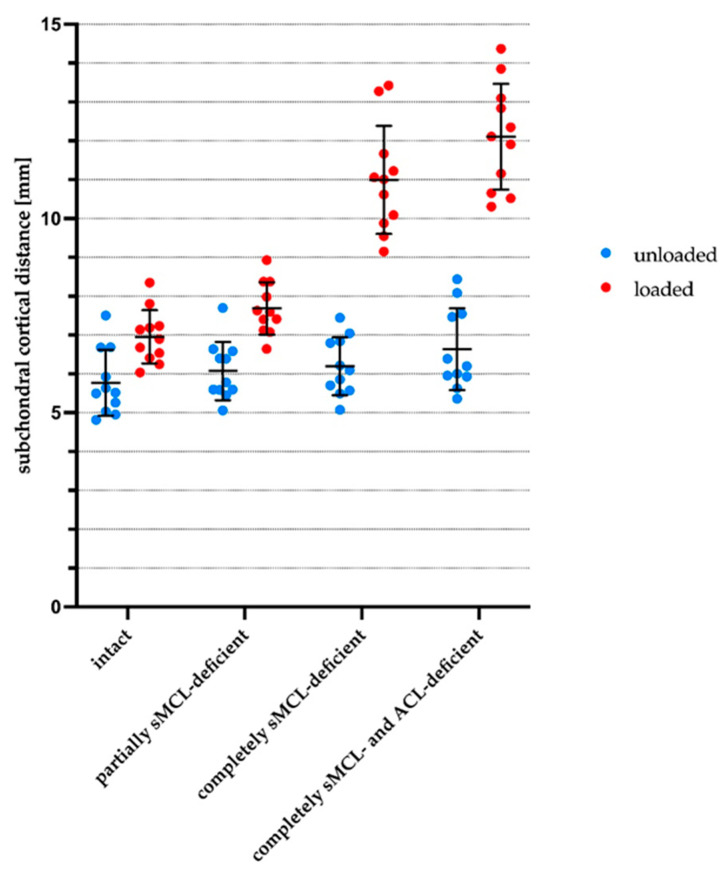
Scatter plots of mean subchondral cortical distances (as determined automatically) as a function of loading and graded knee joint ligament injury. Individual dots indicate mean subchondral cortical distances of each joint, (loading) configuration, and (joint) condition. Horizontal and vertical lines indicate means and standard deviations. Configurations are blue (unloaded) and red (loaded), while conditions are defined below the x-axis. Abbreviations as defined in Figure 6.

Figure 8 provides mean SCD values of the automatic analysis as scatter plots for each individual joint.

Absolute SCD values for the pooled manual 2D and the computed 3D measurements are indicated in Table 2, while the corresponding post-hoc results are outlined in Supplementary Table S1.

Overall, medial compartment opening was largest in the loaded (as compared to the unloaded) configuration, in the sMCL- and ACL-deficient conditions (as compared to the intact or partially sMCL-deficient conditions), and anteriorly (as compared to posteriorly).

In the unloaded configurations, mean SCD values increased slightly with more extensive injury, yet not significantly in most cases. When comparing the loaded versus the unloaded configuration of a single joint condition, mean SCD values increased significantly, irrespective of the joint’s condition. Also, mean SCD values of the loaded configurations of all joint conditions were significantly different.

Quantitatively, these observations translated to the following changes: Intact, loading opened the medial compartment only slightly as indicated by small absolute differences in SCD values that increased from 5.8 ± 0.8 mm (unloaded) to 7.0 ± 0.7 mm (loaded; *p* < 0.001) as an indication of physiological joint laxity. Please note that *p*-values indicate the post-hoc test results of the pair-wise comparison to the intact condition and respective configuration.

Partial sMCL deficiency opened the medial compartment slightly, yet not significantly, to mean SCD values of 6.1 ± 0.7 mm (unloaded, non-significant [ns]), whereas under loading, these changes were significant and mean SCD values increased to 7.7 ± 0.7 mm (loaded, *p* ≤ 0.001).

Complete sMCL deficiency was characterized by distinct increases in medial compartment opening in the loaded configuration and only marginal (yet statistically significant) increases in the unloaded configuration. Mean SCD values increased to values of 11.0 ± 1.4 mm (loaded, *p* ≤ 0.001) and 6.2 ± 0.7 mm (unloaded, *p* < 0.01). In light of the separate and non-overlapping value distributions, possible mean SCD cut-off values for differentiating the completely sMCL-deficient loaded configuration from the completely sMCL-deficient unloaded configuration or from the intact loaded configuration are 7.5 mm to 9.0 mm or 8.4 mm to 9.0 mm, respectively. Of note, the most pronounced increase in SCD values between two consecutive joint conditions and loaded configurations was determined between partial and complete sMCL deficiency, indicating the greatest loss in joint stability after complete sMCL transection.

Additional ACL deficiency induced even larger increases in medial compartment opening that amounted to mean SCD values of 12.1 ± 1.4 mm (loaded, *p* ≤ 0.001) and 6.6 ± 1.1 mm (unloaded, ns).

Regardless of the joint condition, we observed an anteroposterior gradient in the absolute loading-induced differences that were largest for the most anterior SCD locations and smallest for the most posterior SCD locations (Figure 7). The largest loading-induced increases in SCD values originated anteriorly or anteromedially and extended well into the central loadbearing area of the medial compartment.

No significant differences were found between manual 2D reference and computed 3D measurements (Table 2).

## 4. Discussion

The most important finding of this study is that standardized and graded injuries of the sMCL and ACL may be quantitatively assessed using valgus stress MRI, morphologic sequences, and sophisticated image post-processing. By quantifying medial compartment opening in extent and distribution and as a function of loading configuration and joint condition, surrogate imaging markers of instability and functionality may be obtained in a variety of scientific and clinical contexts.

In intact joints, we determined a mean loading-induced medial compartment opening of 1.2 mm (SCD_mean_), which indicated physiological laxity and provided the reference for subsequent comparisons. Not surprisingly, loading induced increases in medial compartment opening that were larger with more widespread injury of the medial stabilizers. The sMCL is the primary restraint to valgus stress and, if injured, brings about medial instability and medial compartment opening of the joint [30,31,32,33,34]. While the functional consequences of complete sMCL tears are clearly defined, the consequences of less severe sMCL injuries such as minor or major strains or partial tears are less clear. While grade-I injuries of the sMCL as encountered in vivo are characterized by high signal adjacent to the ligament without clinical instability [28,35] and cannot be replicated in human cadaveric specimens, our experimental needling approach serves the purpose of evaluating the functional consequences of multiple microscopic fibre tears. Our findings of only marginally increased medial compartment opening (increasing by a mean of 1.6 mm [SCD_mean_] between the unloaded and loaded configurations) are reflective of these changes. Such marginal increases are likely below the human perceptive capacity and may be missed upon clinical examination because physiological side-to-side-differences may be as great as 2 mm [36].

Grade-III injuries indicate complete disruption of the sMCL. We found substantial increases in medial compartment opening under loading with an SCD_mean_ of 4.8 mm, which concurs with previous findings by Warren et al. who reported mean increases of 3.5–5mm of mean medial compartment opening after sectioning of the sMCL in human cadaveric knee joints ([34], between unloaded and loaded, manual loading, 30° and 45° of flexion) and stress sonographic findings by Gruber et al. who found mean increases of 4.4 mm of mean medial compartment opening (between unloaded and loaded, manual loading, 20° of flexion) in patients with complete sMCL tears ([37]). Using stress radiography and human cadaveric knee joints, LaPrade et al. reported a mean increase of 3.2 mm ([11], 10 Nm valgus load, 20° of flexion) after sectioning of the sMCL while Sawant et al. found a substantially larger mean increase of 7.0 mm between the completely sMCL-deficient joint and the intact contralateral joint ([12], manual loading, 10–15° of flexion). Studying human cadaveric joints, too, Grood et al. described an increase of 3 mm ([30], 18 Nm valgus load, 25° of flexion). Overall, complete sMCL deficiency increased medial compartment opening under valgus load by 3 to 7 mm [38], even though the exact amount was largely affected by joint flexion. Extended joints displayed less medial compartment opening than flexed joints and greatest increases were reported across the midrange of knee flexion, i.e., 15°–75° of flexion [33,34,39]. In the present study, joint flexion was standardized to 30° of flexion by using two wedges of 15° inclination each. We consider valgus stress MRI measurements at 30° of flexion particularly beneficial because this joint position does not only exploit the joint’s susceptibility to valgus stress but also allows comparisons of valgus stress MRI to earlier studies (as above) and the definition of dedicated normative values of knee joint instability.

Additional injury of the ACL induced even higher increases in medial compartment opening under loading that averaged 5.5 mm (SCD_mean_) when comparing the unloaded and loaded configurations and 1.1 mm (SCD_mean_) when comparing the loaded configurations of the sMCL-deficient versus the sMCL- and ACL-deficient conditions. In this regard, literature data that assess the functional consequences of additional ACL deficiency are scarce. Using human cadaveric joints, LaPrade et al. reported substantially larger increases of approximately 9 mm after additional injury of the ACL ([11]). Yet, as numerous medial stabilizers such as the meniscofemoral and meniscotibial ligaments as well as the posterior oblique ligament had been transected before the ACL, the comparison is inherently limited due to discrepant sequential sections. Our rationale behind including combined sMCL and ACL deficiencies was guided by this combination’s clinical relevance for the joint’s long-term health, even though the incidence of this particular injury is relatively low [3]. Our findings suggest that ACL injury should be suspected whenever medial compartment opening exceeds the range attributable to an isolated sMCL injury.

Even though our findings confirm the quantitative values of previous studies, it should be noted that these values exhibit inter-study variability which can be attributed to differences in study design, i.e., clinical trials versus cadaveric studies, imaging modalities, i.e., sonography, radiography, and fluoroscopy versus CT and MRI, quantification procedures, i.e., manual versus automatic, and modes of injury, i.e., standardized versus non-standardized.

By combining morphologic MRI sequences and sophisticated image post-processing, we generated 3D joint models and mapped medial compartment opening as subchondral distances between femur and tibia in a spatially resolved manner. Direct comparison of the computed 3D and manual 2D measurements indicated the computed 3D approach is not only valid but may also be used to assess medial joint compartment opening more thoroughly than by single measurements only. Loading-induced medial compartment opening was not uniform across the joint surface as opening was largest anteriorly and smallest posteriorly. This anteroposterior gradient was particularly evident in more widespread injury and may be attributed to anteromedial rotatory instability that leads to pathologic anterior subluxation of the medial tibial plateau relative to the medial femoral condyle under valgus loading [40,41]. The sMCL has recently been found to be the major restraint against anteromedial rotatory instability and its insufficiency (due to transection) results in substantially increased anteromedial and external rotation [42], which distends the medial compartment anteromedially. Earlier approaches that evaluated the joints’ motional changes in one plane only, usually the coronal plane [11,12,37,43], did not consider these underlying complexities and may have fallen short in comprehensively assessing femorotibial flexion, rotation, and translation.

This study has several limitations. First, generalizability is limited since the study was performed on human cadaveric joints only and did not assess dynamic stabilizers such as muscles and tendons [8,31,32]. Younger and more athletic clinical populations were not adequately represented due to the body donor’s advanced age. While to our best knowledge literature data on the association of MCL stiffness and age are not available, corresponding data are available for the ACL. ACL stiffness is much higher in younger adults than in older adults [44,45]. Similarly, osteoarthritis of the joint stiffens the MCL significantly and is another important contributor to medial compartment opening [46]. Overall, these limitations may only be overcome by including younger specimens through alternative sources in future studies. Second, the standardized injury model employed needle stitching and surgical transections and thereby does not fully emulate the complex trauma mechanisms encountered clinically. The mechanisms of grade-I and grade-II sMCL injuries as encountered in vivo are fundamentally different from the injuries induced by experimental needling as used in our study and may not be replicated experimentally. Consequently, our findings of functional consequences of partial sMCL injury may not be extrapolated to clinical grade-I or grade-II sMCL injuries. Moreover, patients may present with additional injuries to the deep medial collateral ligament, the posterior oblique ligament, the posteromedial and anteromedial capsule, the posterior cruciate ligament, and the medial meniscus [8,32,47,48] that were not addressed in this study. It is important to note that this study was focused on providing the respective reference framework of well-defined ligament injuries, while further work must address the impact of different combinations of ligamentous knee injuries as encountered clinically. Third, medial knee instability was not referenced to instrumented laxity measurements or clinical testing. Fourth, we only assessed medial compartment opening at 30° of flexion, which is the clinical reference standard [27,49], while other degrees of flexion were not considered. Fifth, our method of image post-processing relies on time-consuming manual segmentations and, thus, is not (yet) ready for clinical implementation that would require automated segmentations and analyses for expedited workflows. Sixth, because of limited sample size we did not consider (statistically) the body donors’ sex which may be a confounder as women tend to have lower medial joint space widths [16,43] and more severe valgus-laxity [50,51,52]. Seventh, direct clinical translation of our approach is not readily possible. If applied in vivo, the layout of the loading device and the orientation of the two quarter-pipe wedges would translate to the patient being scanned in the prone position with the ipsilateral hip and foot as well as the pelvis, abdomen, and thorax being elevated to some extent. While principally feasible (yet most likely uncomfortable) in small patients, this setup will not work for tight MRI bores and large patients. Consequently, clinical translation would require revision of the loading unit, including the wedges, to allow patient loading at 30° of flexion and in the supine position. Besides joint position and its mechanical fixation, additional aspects such as patient comfort, device safety and operability, loading reproducibility, and measurement validity will need to be addressed in future clinical studies.

## 5. Conclusions

In conclusion, this study indicated that valgus stress MRI, if complemented by dedicated image post-processing, is suitable to quantify medial compartment opening as a functional imaging surrogate of medial knee joint instability and beyond mere morphology. This study also provides normative data of medial compartment opening as a function of loading and injury, which may be used to prospectively assist in differential diagnosis, therapeutic decision-making, and monitoring of treatment outcomes of medial knee joint injuries once validation in clinical trials is completed.

## Figures and Tables

**Table 1:** Acquisition Parameters of the MRI sequences.

	PDw fs	PDw fs	PDw fs	T1w	T2w	T2w
**Sequence Type**	2D TSE	2D TSE	2D TSE	2D TSE	2D TSE	2D TSE
**Orientation**	ax	sag	cor	cor	ax	cor
**Type of fat saturation**	SPAIR	SPAIR	SPAIR	n/a	n/a	n/a
**Repetition Time [ms]**	4776	7595	4495	671	3283	3000
**Echo time [ms]**	30	30	30	9	80	80
**Turbo spin-echo factor**	13	15	13	3	14	14
**Field of view [mm]**	160 × 160	164 × 144	160 × 160	160 × 160	160 × 160	160 × 160
**Acquisition matrix [pixels]**	400 × 312	352 × 256	400 × 300	368 × 317	352 × 295	352 × 297
**Reconstruction matrix [pixels]**	512 × 512	512 × 512	512 × 512	448 × 448	512 × 512	512 × 512
**Scan percentage [%]**	79.4	79.3	79.4	86.4	85.0	85.0
**Flip angle [°]**	90	90	90	90	90	90
**Number of signal averages**	1	1	1	1	1	1
**Slices**	33	40	31	30	40	30
**Slice Thickness/Gap [mm]**	3.0/0.3	3.0/0.3	3.0/0.3	3.0/0.3	1.5/0.0	1.5/0.3
**Duration [min:s]**	03:59	05:19	04:39	06:40	03:17	03:42

Abbreviations: PDw—proton density-weighted, T1w—T1-weighted, T2w—T2-weighted, fs—fat-saturated, TSE—turbospin echo, cor—coronal, ax—axial, sag—sagittal, SPAIR—Spectral Attenuated Inversion Recovery, n/a—not applicable.

**Table 2:** Manual 2D and computed 3D measures of medial compartment opening as a function of valgus loading and graded knee joint ligament injury. Manual 2D measurements of subchondral cortical distances (SCDs) were performed at six pre-specified locations (SCD_ap1_ to SCD_ap3_ and SCD_ml1_ to SCD_ml3_) by two readers. Because of excellent inter-reader agreement, measurements were pooled. SCD_mean_ indicates the averaged SCD values over the entire medial compartment. Mean ± standard deviation [mm]. Repeated measures two-way ANOVA was used to assess the SCD values of each location longitudinally and as a function of configuration (i.e., unloaded [UL], loaded [LO]) and condition (i.e., intact, partially, and completely sMCL-deficient, and completely sMCL- and ACL-deficient); respective *p*-values are indicated line-wise (‡). Paired Student’s *t*-test was used to comparatively evaluate SCD values of manual 2D measurements and computed 3D measurements; respective *p*-values are indicated column-wise (†). Significant differences are highlighted in bold-typed *p*-values and corresponding post-hoc results are detailed in Supplementary Table S1. Abbreviations as defined in Figure 6.

		Intact		Partially sMCL-Deficient		Completely sMCL-Deficient		Completely sMCL- and ACL-deficient		*p*-Value (‡)
		UL	LO	UL	LO	UL	LO	UL	LO	
**Manual 2D** **Measurements**	SCD_ml1_	3.7 ± 0.7	4.8 ± 1.0	3.9 ± 0.7	5.6 ± 1.0	4.0 ± 0.7	9.2 ± 1.4	4.7 ± 1.0	10.6 ± 1.4	**<0.001**
	SCD_ml2_	3.7 ± 0.6	4.7 ± 0.9	3.9 ± 0.7	5.3 ± 0.8	4.2 ± 0.6	8.7 ± 1.5	4.5 ± 0.9	9.6 ± 1.5	
	SCD_ml3_	4.8 ± 0.9	5.9 ± 0.9	4.9 ± 0.9	6.5 ± 1.0	5.3 ± 0.9	10.0 ± 1.5	5.4 ± 1.3	10.6 ± 1.6	
	SCD_ap1_	6.2 ± 1.4	7.6 ± 1.5	6.4 ± 1.2	8.3 ± 1.5	6.4 ± 1.5	11.9 ± 2.1	7.1 ± 1.4	14.4 ± 2.2	
	SCD_ap2_	3.9 ± 0.5	4.8 ± 0.7	4.1 ± 0.4	5.4 ± 0.8	4.4 ± 0.6	8.9 ± 1.6	4.8 ± 0.8	9.8 ± 1.5	
	SCD_ap3_	7.4 ± 1.1	8.0 ± 1.2	7.8 ± 1.2	8.4 ± 1.1	8.0 ± 1.3	10.8 ± 1.4	7.9 ± 1.5	10.8 ± 1.9	
**Computed 3D** **Measurements**	SCD_mean_	5.8 ± 0.8	7.0 ± 0.7	6.1 ± 0.7	7.7 ± 0.7	6.2 ± 0.7	11.0 ± 1.4	6.6 ± 1.1	12.1 ± 1.4	**<0.001**
	SCD_ml1_	3.7 ± 0.8	4.9 ± 0.8	4.1 ± 0.6	5.7 ± 0.8	4.1 ± 0.7	9.4 ± 1.3	4.7 ± 0.9	10.7 ± 1.5	
	SCD_ml2_	3.9 ± 0.7	4.9 ± 0.9	4.1 ± 0.7	5.5 ± 1.0	4.3 ± 0.6	8.9 ± 1.4	4.6 ± 0.8	9.9 ± 1.6	
	SCD_ml3_	5.0 ± 0.9	5.9 ± 0.9	5.3 ± 0.8	6.4 ± 0.8	5.4 ± 0.9	10.0 ± 1.4	5.4 ± 1.0	10.6 ± 1.3	
	SCD_ap1_	5.7 ± 1.0	7.6 ± 1.3	5.7 ± 1.1	8.1 ± 1.4	6.0 ± 1.3	12.2 ± 1.8	6.7 ± 1.9	14.4 ± 2.0	
	SCD_ap2_	3.8 ± 0.8	4.9 ± 0.9	4.1 ± 0.7	5.5 ± 1.0	4.2 ± 0.6	8.8 ± 1.4	4.7 ± 0.8	9.8 ± 1.5	
	SCD_ap3_	7.1 ± 1.4	7.8 ± 1.0	7.9 ± 1.3	8.3 ± 1.7	7.7 ± 1.6	10.8 ± 1.9	7.7 ± 1.1	10.8 ± 2.3	
***p*-value (†)**	SCD_ml1_	0.871	0.798	0.156	0.748	0.785	0.314	0.976	0.397	
	SCD_ml2_	0.180	0.061	0.269	0.051	0.062	0.056	0.102	0.051	
	SCD_ml3_	0.082	0.770	0.103	0.333	0.277	0.924	0.872	0.989	
	SCD_ap1_	0.126	0.984	0.101	0.233	0.168	0.347	0.187	0.836	
	SCD_ap2_	0.827	0.668	0.960	0.846	0.086	0.363	0.171	0.856	
	SCD_ap3_	0.356	0.494	0.635	0.677	0.372	0.832	0.554	0.839	

## Data Availability

The data not contained in the manuscript or Supplementary Material is available from the corresponding author upon reasonable request.

## Supplementary Materials

The following are available online at https://www.mdpi.com/article/10.3390/diagnostics11081433/s1, Supplementary Figure S1: Visualization of the 3D joint model and subchondral cortical distances. Supplementary Table S1: Post-hoc details of pairwise comparisons of absolute manual 2D and computed 3D measurements of subchondral cortical distances (SCDs).
