# Supplementary material for: Comprehensive Assessment of Medial Knee Joint Instability by Valgus Stress MRI
Source: Diagnostics (Basel). 2021 Aug 9;11(8):1433. doi: 10.3390/diagnostics11081433 (PMC8392372; doi:10.3390/diagnostics11081433)
Supplement: Supplementary file 1 [file diagnostics-11-01433-s001.zip › diagnostics-1324291-supplementary.pdf]

Supplementary Material

Title: Comprehensive Assessment of Medial Knee Joint Instability by Valgus Stress MRI

Authors: Malin Ciba, Eva-Maria Winkelmeyer, Justus Schock, Philipp Schad, Niklas Kotowski, Teresa Nolte, Lena Marie Wollschläger, Matthias Knobe, Andreas Prescher, Christiane Kuhl, Daniel Truhn, Sven Nebelung

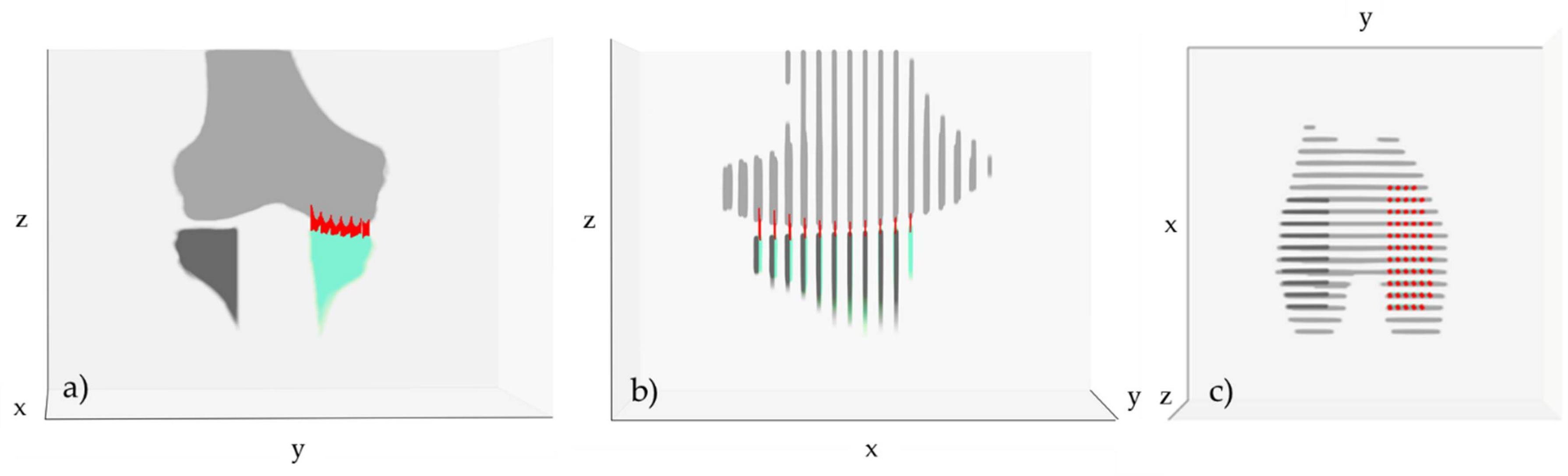

Supplementary Figure S1: Visualization of the 3D joint model and subchondral cortical distances. Based on manual segmentations of femur (light grey) and tibia (dark grey [lateral condyle]; turquoise [medial condyle]), 3D joint models were implemented for each joint, configuration, and condition. Subchondral cortical distances were determined at closely spaced intervals along the tibial plateau. The 3D model was considered as a Cartesian coordinate system in which the x-axis indicates the anteroposterior, the y-axis the mediolateral, and the z-axis the craniocaudal dimension. Accordingly, the y-z-plane represents the coronal plane (a), the x-z-plane the sagittal plane (b), and the x-y-plane the axial plane of the acquired MR images (c). A grid with fixed spacing of 3.3 mm (along x) and 3.5 mm (along y) was defined for the medial compartment of each joint, configuration, and condition. Originating from each grid point, represented by red dots (c), grid lines, represented by red lines (a, b), were computed parallel to the z-axis, and measured in length between both cortical bone outlines. Sliced appearance of femur and tibia (b, c) is due to interslice gaps after reconstruction.

Supplementary Table S1: **Post-hoc details of pairwise comparisons of absolute manual 2D and computed 3D measurements of subchondral cortical distances (SCDs).** The manual 2D measurements of the two readers were pooled. Following repeated measures two-way ANOVA, Tukey's multiple comparisons test was performed to assess significant differences between selected loading configurations and joint conditions. This selection was performed to reduce the number of comparisons. The family-wise alpha threshold was set to p≤.01. ns – non-significant, otherwise please refer to **Table 2** for details on abbreviations.

	**Tukey's post-hoc test**	**SCD$_{mean}$**	**SCD$_{ml1}$**	**SCD$_{ml2}$**	**SCD$_{ml3}$**	**SCD$_{ap1}$**	**SCD$_{ap2}$**	**SCD$_{ap3}$**
**Manual 2D Measurements**	Intact UL vs. intact LO	-	0.003	ns	ns	0.003	0.004	ns
	intact UL vs. partially sMCL-deficient UL	-	ns	ns	ns	ns	ns	ns
	intact UL vs. completely sMCL-deficient UL	-	ns	ns	ns	ns	ns	ns
	intact UL vs. completely sMCL- and ACL-deficient UL	-	0.008	ns	ns	ns	ns	ns
	intact LO vs. partially sMCL-deficient LO	-	0.002	0.003	ns	ns	<0.001	ns
	intact LO vs. completely sMCL-deficient LO	-	<0.001	<0.001	<0.001	<0.001	<0.001	<0.001
	intact LO vs. completely sMCL- and ACL-deficient LO	-	<0.001	<0.001	<0.001	<0.001	<0.001	0.001
	partially sMCL-deficient UL vs. partially sMCL-deficient LO	-	<0.001	0.001	0.001	<0.001	<0.001	ns
	partially sMCL-deficient UL vs. completely sMCL-deficient UL	-	ns	ns	ns	ns	ns	ns
	partially sMCL-deficient UL vs. completely sMCL- and ACL-deficient UL	-	ns	ns	ns	ns	ns	ns
	partially sMCL-deficient LO vs. completely sMCL-deficient LO	-	<0.001	<0.001	<0.001	<0.001	<0.001	<0.001
	partially sMCL-deficient LO vs. completely sMCL- and ACL-deficient LO	-	<0.001	<0.001	<0.001	<0.001	<0.001	0.002
	completely sMCL-deficient UL vs. completely sMCL-deficient LO	-	<0.001	<0.001	<0.001	<0.001	<0.001	<0.001
	completely sMCL-deficient UL vs. completely sMCL- and ACL-deficient UL	-	ns	ns	ns	ns	ns	ns
	completely sMCL-deficient LO vs. completely sMCL- and ACL-deficient LO	-	0.007	0.007	ns	0.003	0.003	ns
	completely sMCL- and ACL-deficient UL vs. completely sMCL- and ACL-deficient LO		<0.001	<0.001	<0.001	<0.001	<0.001	<0.001
**Computed 3D Measurements**	Intact UL vs. intact LO	<0.001	0.001	0.005	ns	<0.001	0.006	ns
	intact UL vs. partially sMCL-deficient UL	ns	ns	ns	ns	ns	ns	ns
	intact UL vs. completely sMCL-deficient UL	<0.01	ns	ns	ns	ns	ns	ns
	intact UL vs. completely sMCL- and ACL-deficient UL	ns	0.007	ns	ns	ns	ns	ns
	intact LO vs. partially sMCL-deficient LO	<0.001	0.002	0.004	0.004	ns	0.002	ns
	intact LO vs. completely sMCL-deficient LO	<0.001	<0.001	<0.001	<0.001	<0.001	<0.001	0.006
	intact LO vs. completely sMCL- and ACL-deficient LO	<0.001	<0.001	<0.001	<0.001	<0.001	<0.001	ns
	partially sMCL-deficient UL vs. partially sMCL-deficient LO	<0.001	<0.001	0.004	ns	0.003	0.004	ns

partially sMCL-deficient UL vs. completely sMCL-deficient UL	ns	ns	ns	ns	ns	ns	ns
partially sMCL-deficient UL vs. completely sMCL- and ACL-deficient UL	ns	ns	ns	ns	ns	ns	ns
partially sMCL-deficient LO vs. completely sMCL-deficient LO	<0.001	<0.001	<0.001	<0.001	<0.001	<0.001	ns
partially sMCL-deficient LO vs. completely sMCL- and ACL-deficient LO	<0.001	<0.001	<0.001	<0.001	<0.001	<0.001	ns
completely sMCL-deficient UL vs. completely sMCL-deficient LO	<0.001	<0.001	<0.001	<0.001	<0.001	<0.001	<0.001
completely sMCL-deficient UL vs. completely sMCL- and ACL-deficient UL	ns	ns	ns	ns	ns	ns	ns
completely sMCL-deficient LO vs. completely sMCL- and ACL-deficient LO	0.001	0.008	0.007	ns	ns	0.005	ns
completely sMCL- and ACL-deficient UL vs. completely sMCL- and ACL-deficient LO	<0.001	<0.001	<0.001	<0.001	<0.001	<0.001	0.001
